# Effects of Perfluorooctanoic Acid on Gut Microbiota and Microbial Metabolites in C57BL/6J Mice

**DOI:** 10.3390/metabo13060707

**Published:** 2023-05-30

**Authors:** Bei Gao, Lixia Chen, Weichen Xu, Jinjun Shan, Weishou Shen, Nan Gao

**Affiliations:** 1 School of Marine Sciences, Nanjing University of Information Science and Technology, Nanjing 210044, China; wintergb@hotmail.com (B.G.); 201983450063@nuist.edu.cn (L.C.); 2 Key Laboratory of Hydrometeorological Disaster Mechanism and Warning of Ministry of Water Resources, Nanjing University of Information Science and Technology, Nanjing 210044, China; 3 Medical Metabolomics Center, Jiangsu Key Laboratory of Pediatric Respiratory Disease, Institute of Pediatrics, Nanjing University of Chinese Medicine, Nanjing 210023, China; xuweichen@njucm.edu.cn (W.X.); jshan@njucm.edu.cn (J.S.); 4 School of Environmental Science and Engineering, Nanjing University of Information Science and Technology, Nanjing 210044, China; wsshen@nuist.edu.cn; 5 Jiangsu Key Laboratory of Atmospheric Environment Monitoring and Pollution Control, Collaborative In-novation Center of Atmospheric Environment and Equipment Technology, Nanjing 210044, China; 6 Institute of Soil Health and Climate-Smart Agriculture, Nanjing University of Information Science and Technology, Nanjing 210044, China; 7 School of Biological and Pharmaceutical Engineering, Nanjing Tech University, Nanjing 211816, China

**Keywords:** gut microbiome, tryptophan derivatives, glycodeoxycholic acid, beta-hyodeoxycholic acid, gamma-muricholic acid

## Abstract

Perfluorooctanoic acid (PFOA) represents an increasing public health concern due to its persistence in the environment and its toxic effects. The gut microbiota is known to produce various metabolites that assist the host to maintain metabolic homeostasis. However, few studies have explored the effects of PFOA on gut-microbiota-related metabolites. In the present study, male C57BL/6J mice were exposed to 1 ppm of PFOA in drinking water for four weeks and integrative analysis of the gut microbiome and metabolome was performed to reveal the health effects of PFOA. Our results showed that PFOA disturbed both the gut microbiota composition and the metabolic profiles of the feces, serum, and liver in mice. A correlation was found between *Lachnospiraceae UCG004*, *Turicibacter*, *Ruminococcaceae*, and different fecal metabolites. Significant alterations of gut-microbiota-related metabolites were induced by PFOA exposure, including bile acids and tryptophan metabolites such as 3-indoleacrylic acid and 3-indoleacetic acid. The findings of this study are helpful to improve the understanding of the health effects of PFOA, which might be mediated through the gut microbiota and its related metabolites.

## 1. Introduction

Perfluorooctanoic acid (PFOA) is a manufactured per- and poly-fluoroalkyl substance (PFAS) that is extensively applied in fire-fighting foams, surfactants, surface-protective materials, and other commercial products. In the United States, the existence of PFOA has been found in more than 100 public water systems [1]. PFOA is listed as a Persistent Organic Pollutant in the Stockholm Convention, and its production has been phased out in many countries. However, PFOA is highly persistent in the environment and still threatens both the environment and human health. Humans are exposed to PFOA mainly through the consumption of contaminated drinking water and food, followed by dermal absorption and inhalation [2]. PFOA was detected in human plasma in a Danish mother–child cohort consisting of 738 pregnant women and their children [3], and in participants who were enrolled in a Diabetes Prevention Program trial in the US [4]. PFOA is considered to be present in the serum of virtually all people in industrialized countries [5]. The half-life of PFOA in humans has been proposed to be 0.5–1.5 years based on human observational research and clinical studies [6]. In October 2021, the US Environmental Protection Agency (EPA) released the PFAS Strategic Roadmap to protect human health and the environment from the hazards of PFAS. The US EPA’s lifetime drinking water health advisory limit for PFOA is 0.004 parts per trillion (ppt). Various health effects have been linked to PFOA exposure. For instance, PFOA stimulated cell viability, inhibited free radical production, and disrupted the redox status in swine granulosa cells, with the potential impairment of reproductive function, which requires an adequate oxidative balance [7].

PFOA can induce gut microbial dysbiosis in mice [8,9,10]. The gut microbiota is composed of microorganisms living in the gastrointestinal tract, which is an integral part of the host. The composition of the gut microbiota is dynamic and can be affected by many factors, including environmental contaminants, such as pesticides [11,12], heavy metals [13,14], and personal care products [15]. Dysbiosis of the gut microbiota is associated with various diseases, such as ulcerative colitis [16]. Higher serum levels of PFOA have been found in patients with ulcerative colitis compared to control subjects [5]. The gut microbiota exerts a remarkable influence on the health and disease states of the host through the production or modification of various metabolites. The metagenome of the gut microbiota has a vast capacity to carry out a wide range of metabolic processes, such as the production of short chain fatty acids, the production of tryptophan derivatives, and the transformation of primary bile acids to secondary bile acids. These gut-microbiota-related metabolites play a key role in maintaining the metabolic homeostasis of the host.

As an essential amino acid, tryptophan can be transformed into different tryptophan metabolites by the gut microorganisms, such as 3-indoleacrylic acid and 3-indoleacetic acid. Tryptophan metabolites can activate signaling pathways and control the homeostasis and the function of immune cells through transcription factors such as the aryl hydrocarbon receptor (AhR) [17]. Primary bile acids are synthesized in host hepatocytes from cholesterol, which enter the gastrointestinal tract and are modified by the gut microbiota into secondary bile acids. Bile acids are recognized as signaling factors and metabolic integrators, and they have become therapeutic targets for diseases such as metabolic disorder [18]. The gut microbiota can influence the bile acid pool; meanwhile, the bile acid pool can modulate the community structure of the gut microbiota [19].

The gut and gut microbiota have a bidirectional relationship with the liver, known as the ‘gut–liver axis’. Gut-derived metabolites and products can be transported to the liver through the portal vein. Meanwhile, the liver secretes bile acids into the intestine to facilitate the digestion of fat and fat-soluble nutrients. The gut microbiota plays a crucial role in maintaining the homeostasis of the gut–liver axis and the bile acid pool. Gut-microbiota-derived metabolites are also critical in the pathogenesis of liver diseases, such as secondary bile acids [20] and trimethylamine N-oxide (TMAO) [21]. PFOA-induced liver damages can be alleviated by the supplementation of lactic acid bacteria or gastrodin [22,23].

The gut microbiota can influence the metabolic homeostasis of the host by producing gut-microbiota-related metabolites. However, an integrative analysis of the gut microbiota and metabolome disturbed by PFOA exposure has not been carried out. In this study, we performed 16S rRNA gene sequencing, in combination with untargeted metabolomics, analysis on fecal, serum, and liver samples from PFOA-treated C57BL/6J mice to reveal the effects of PFOA on the gut microbiota and its related metabolites. The findings of this study are helpful to improve the understanding of the toxic effects of PFOA.

## 2. Materials and Methods

### 2.1. Animals

Four-week-old specific pathogen-free C57BL/6J male mice were purchased from Jackson Laboratory (Bar Harbor, ME, USA). Mice consumed drinking water *ad libitum*. The animal facility at the University of Georgia was maintained at 22 °C and 40−70% humidity, under a 12:12 h light:dark cycle. Before the PFOA exposure experiment, five mice were housed in one cage, and the mice changed cages randomly once a week for a four-week period. PFOA exposure began when the mice were eight weeks old and the exposure period lasted four weeks. At the beginning of the experiment, mice were assigned to the PFOA exposure group or control group randomly (*n* = 10 mice in each group), with one mouse in one cage. PFOA (Sigma-Aldrich, St. Louis, MO, USA) was administered to mice through drinking water. The final concentration of PFOA in drinking water was 1 part per million (ppm). The dose was chosen based on our previous study [24]. Control mice consumed drinking water without the addition of PFOA. The animal protocol was approved by the Institutional Animal Care and Use Committee, University of Georgia (protocol code: A2014 10-014-Y2-A1).

### 2.2. Metabolite Extraction

The extraction of metabolites was performed as described previously [25]. Briefly, 20 μL of each serum sample was extracted with 225 μL methanol. The samples were vortexed for 10 s. Then, 750 μL methyl tert-butyl ether (MTBE) and 188 μL water were added to the above samples to induce phase separation. The samples were shaken at 4 °C for 6 min, followed by centrifugation at 14,000× *g* for 2 min. The extraction solvent was degassed and pre-cooled at −20 °C. The upper non-polar phase (350 μL) was collected and evaporated to complete dryness using a Labconco Centrivap cold trap concentrator (Labconco, Kansas, MO, USA). For liver and fecal samples, 225 μL methanol and 750 μL MTBE were added to 10 mg of each sample. The samples were homogenized using a GenoGrinder 2010 (SPEX SamplePrep, Metuchen, NJ, USA) at a speed of 1500 rpm for 30 s and then vortexed and shaken at 4 °C for 6 min. Then, 188 μL water was added to the samples, followed by centrifugation at 14,000× *g* for 2 min. The upper non-polar phase (350 μL) was collected and evaporated to complete dryness. Dried samples were then resuspended in 60 μL 4:1 acetonitrile and water (*v*/*v*) with internal standards. Samples were vortexed, followed by sonication for 5 min and centrifugation at 14,000× *g* for 2 min. The supernatant was collected for LC-MS analysis.

### 2.3. Untargeted Metabolomics Profiling

Untargeted metabolomics profiling was performed as described previously [25]. The Vanquish UHPLC system (Thermo Scientific, Waltham, MA, USA) was used for metabolites separation with an Acquity UPLC BEH Amide column (150 mm × 2.1 mm × 1.7 μm), which was coupled to an Acquity VanGuard BEH Amide pre-column (5 mm × 2.1 mm × 1.7 μm, Waters, Milford, MA, USA). LC-MS-grade water (100%) with 10 mM ammonium formate and 0.125% formic acid (Sigma-Aldrich, St. Louis, MO, USA) was used as mobile phase A. Meanwhile, 95:5 acetonitrile: water (*v*/*v*) containing 10 mM ammonium formate and 0.125% formic acid were used as mobile phase B. The gradient began with 100% B at 0–2 min, 70% B at 7.7 min, 40% B at 9.5 min, and 30% B at 10.25 min, followed by 100% B at 12.75–16.75 min. The flow rate was kept at 0.4 mL/min. Spectra were collected in electrospray ionization (ESI) positive mode using Q-Exactive HF (Thermo Scientific, Waltham, MA, USA). The mass range was set from *m*/*z* 60 to 900 with data-dependent mode for MS/MS spectra acquisition. Untargeted metabolomics raw data were first converted into ABF files using the ABF converter (https://www.reifycs.com/AbfConverter/ (accessed on 6 July 2022)). MS-DIAL version 5.1.0.1 was used for the identification and quantification of metabolites [26]. The Mass Spectral Feature List Optimizer (MS-FLO) was used to improve the quality of feature lists by flagging features for further data curation [27]. Retention time *m*/*z* libraries and Fiehn HILIC MS/MS spectra databases in MassBank of North America were used for the identification of metabolites.

### 2.4. 16S rRNA Gene Sequencing

The total DNA was extracted from fecal samples using the PowerSoil^®^ DNA isolation kit (MO BIO Laboratories, Carlsbad, CA, USA), following the manufacturer’s instructions. The 515F and 806R primers were used for amplification of the V4 region of the 16S rRNA gene, followed by normalization and barcoding. DNA was sequenced on the Illumina MiSeq v2 kit with 500 cycles. The 16S rRNA gene sequencing data were analyzed using Quantitative Insights into Microbial Ecology (QIIME) II [28]. Briefly, raw sequencing data were denoised via DADA2, followed by operational taxonomic unit (OTU) selection. SILVA (version 138), trained by the scikit-learn Naïve-Bayes-based classifier, was used as a database.

### 2.5. Statistical Analysis

Linear discriminant analysis effect size (LEfSe) was used to determine the bacterial genera that were most likely to explain the differences between the PFOA and control groups [29]. For the comparison of metabolites, the Mann−Whitney U test was used to calculate the *p*-values between the PFOA and control groups. The false discovery rate was used to correct the multiple comparisons. Metabolites with a *p*-value < 0.05 were considered significant. Partial least squares discriminant analysis (PLS-DA) and pathway enrichment analysis were performed using MetaboAnalyst 5.0 [30]. MixOmics was used for the integrative analysis of gut microbiota and fecal metabolites—this is an integrative framework for the multivariate analysis of omics data [31].

## 3. Results

### 3.1. PFOA Altered the Composition of the Gut Microbiota

**Figure 1:**
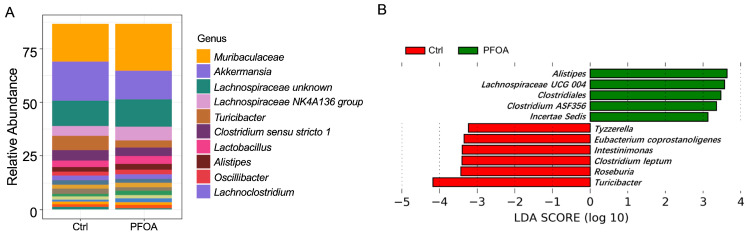
PFOA altered the composition of the gut microbiota. (**A**) Abundant microbial genera detected in control and PFOA-treated mice. (**B**) Microbial genera significantly altered by PFOA exposure, as revealed by LEfSe analysis.

A total of 78 bacterial genera were detected in our study, as revealed by 16S rRNA sequencing. The most abundant genera are shown in Figure 1A. LEfSe analysis showed that five bacterial genera were enriched in PFOA-treated mice, namely *Alistipes*, *Lachnospiraceae UCG 004*, *Clostridiales*, *Clostridium ASF356*, and *Incertae sedis* (Figure 1B). Meanwhile, six bacterial genera were reduced in the PFOA-treated group, namely *Tyzzerella*, *Eubacterium coprostanoligenes*, *Intestinimonas*, *Clostridium leptum*, *Roseburia*, and *Turicibacter* (Figure 1B).

### 3.2. Correlation between Gut Microbiota and Fecal Metabolites

**Figure 2:**
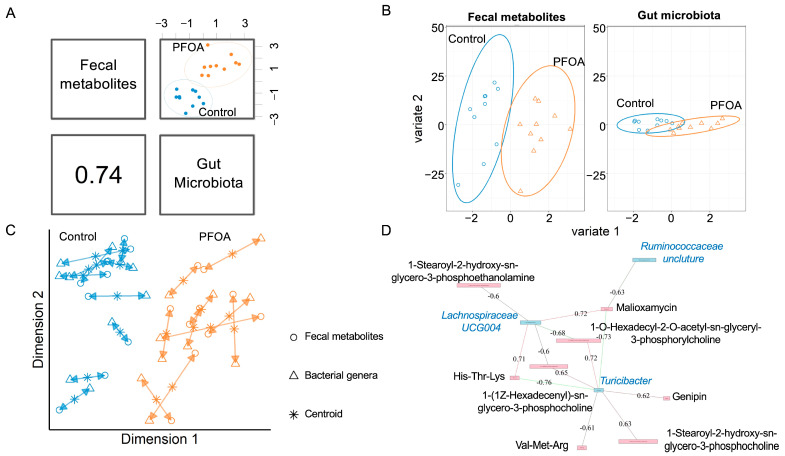
Correlated fecal metabolites and gut microbiota. (**A**) Overall correlation between fecal metabolites and gut bacteria. (**B**) sPLS-DA sample projections for fecal metabolites (**left** panel) and gut microbiota (**right** panel). (**C**) Integrative analysis of fecal metabolites and gut microbiota. Each sample corresponds to one double-headed arrow. Short arrow: strong agreement between two datasets; long arrow: disagreement between two datasets. (**D**) Correlations between gut microbiota and fecal metabolites. Cut-off: 0.6; pink line: positive correlation; green line: negative correlation. Pink box: metabolites; blue box: bacteria. Black font: metabolites; blue font: bacteria.

We performed an integrative analysis of the gut microbiota and fecal metabolites using MixOmics. The overall correlation between the gut microbiota and fecal metabolites was 0.74 (Figure 2A). For both fecal metabolites and bacterial genera, the PFOA-treated group was separated from the control group (Figure 2B, Supplementary Figure S1). The agreement between the fecal metabolites and bacterial genera is shown in Figure 2C. Three bacterial genera showed correlations with eight fecal metabolites (Figure 2D).

### 3.3. PFOA Induced Changes in Fecal Metabolites

**Figure 3:**
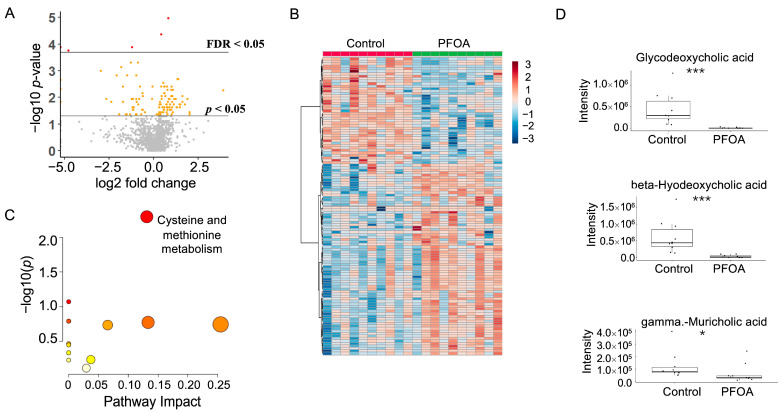
PFOA-disturbed fecal metabolites. (**A**) Volcano plot. Red dot: FDR < 0.05; Yellow dot: *p*-value < 0.05; Grey dot: *p*-value > 0.05. (**B**) Heatmap of fecal metabolites. (**C**) Pathway enrichment analysis of fecal metabolites. Red bubble with label: *p*-value < 0.05. (**D**) Boxplots of fecal glycodeoxycholic acid, beta-hyodeoxycholic acid, and gamma-muricholic acid. *: *p*-value < 0.05; ***: *p*-value < 0.001.

After PFOA exposure, a total of 120 metabolites showed a *p*-value < 0.05, among which five metabolites showed a false discovery rate (FDR) < 0.05 (Figure 3A). Hierarchical clustering of the significant metabolites is shown in Figure 3B. Pathway enrichment analysis revealed that cysteine and methionine metabolism was altered by PFOA exposure (Figure 3C). Notably, three fecal bile acids were significantly reduced by PFOA exposure, namely glycodeoxycholic acid, beta-hyodeoxycholic acid, and gamma-muricholic acid (Figure 3D).

### 3.4. PFOA Induced Changes in Serum Metabolites

**Figure 4:**
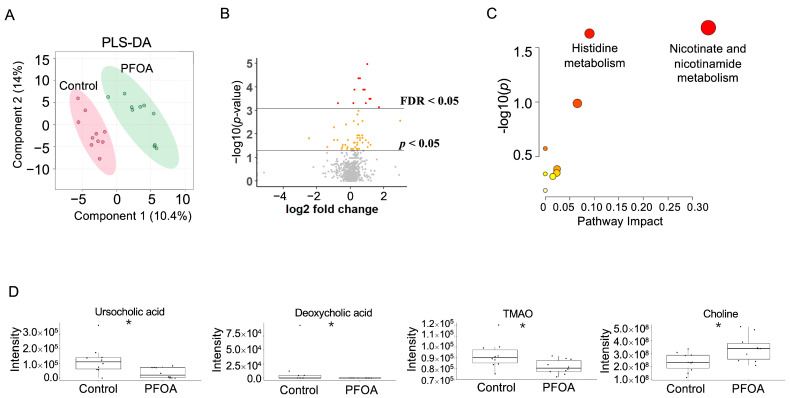
PFOA-disturbed serum metabolites. (**A**) PLS-DA plot for serum metabolites. (**B**) Volcano plot. Red dot: FDR < 0.05; Yellow dot: *p*-value < 0.05; Grey dot: *p*-value > 0.05. (**C**) Pathway enrichment analysis of serum metabolites. Red bubble with label: *p*-value < 0.05. (**D**) Boxplots of serum ursocholic acid, deoxycholic acid, TMAO, and choline. *: *p*-value < 0.05.

The PFOA-treated group and control group were well separated in the PLS-DA plot (Figure 4A). A total of 59 serum metabolites showed a *p*-value < 0.05, among which 10 metabolites showed an FDR < 0.05 (Figure 4B). Pathway enrichment analysis revealed that nicotinate and nicotinamide metabolism and histidine metabolism were altered by PFOA exposure (Figure 4C). Two bile acids, ursocholic acid and deoxycholic acid, were significantly decreased in the PFOA-treated group, in line with the decrease in TMAO and the increase in choline caused by PFOA exposure (Figure 4D).

### 3.5. PFOA Induced Changes in Liver Metabolites

**Figure 5:**
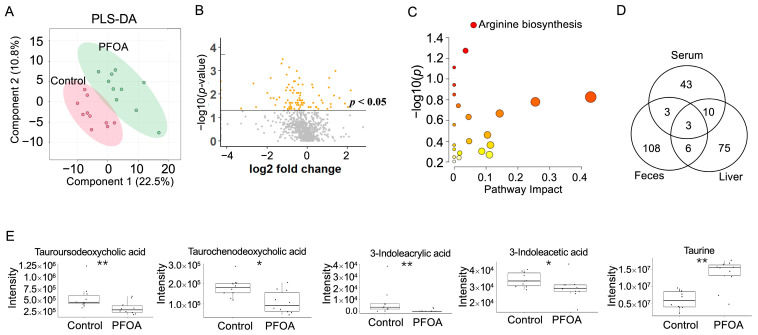
PFOA-disturbed liver metabolites. (**A**) PLS-DA plot for liver metabolites. (**B**) Volcano plot. Yellow dot: *p*-value < 0.05; Grey dot: *p*-value > 0.05. (**C**) Pathway enrichment analysis of serum metabolites. Red bubble with label: *p*-value < 0.05 (**D**) Venn diagram for significantly altered metabolites in feces, serum, and liver. (**E**) Boxplots of liver tauroursodeoxycholic acid, taurochenodeoxycholic acid, 3-indoleacrylic acid, 3-indoleacetic acid, and taurine. *: *p*-value < 0.05; **: *p*-value < 0.01.

PLS-DA analysis of liver metabolites showed that the PFOA-treated group was separated from the control group (Figure 5A). A total 94 liver metabolites showed *p*-values < 0.05 after PFOA exposure (Figure 5B). Pathway enrichment analysis showed that arginine biosynthesis was significantly altered (Figure 5C). Three metabolites were found to be significant in the liver, serum, and feces, namely 1-(1Z-hexadecenyl)-sn-glycero-3-phosphocholine, decanoyl-L-carnitine, and 1-hexadecyl-sn-glycero-3-phosphocholine (Figure 5D). Two bile acids, tauroursodeoxycholic acid and taurochenodeoxycholic acid, were reduced by PFOA exposure, along with decreases in two tryptophan metabolites, 3-indoleacrylic acid and 3-indoleacetic acid, and an increase in taurine (Figure 5E).

## 4. Discussion

The carbon–fluorine bond makes PFOA resistant to photolysis and hydrolysis, which leads to the persistence of PFOA in the environment. A positive correlation was found between plasma PFAS concentrations and the dietary intake of freshwater fish, marine fish, crab, and shrimp in 933 reproductive-aged women in Shanghai, China [32]. Early-life exposure to PFOA is a key factor in the serum concentrations in children [33]. In a study that examined the relationship between PFAS and chronic inflammation and oxidative stress markers within the National Health and Nutrition Examination Survey (NHANES) from 2005 to 2012 (*n* = 6652), a percentage change in PFOA was significantly associated with percentage increases in lymphocyte counts, serum iron, and serum total bilirubin [34]. The kidney and testicular cancer risk increases per 10 ng/mL elevation in the serum PFOA concentration [35]. In addition, the serum PFOA concentration was positively associated with hepatocellular damage marker alanine aminotransferase (ALT) in 47,092 adults in the C8 Health Project [36]. Serum PFOA was also positively associated with immunoglobulin G, anti-cyclic citrullinated peptide antibody, and rheumatoid factors [37].

As a serious and widespread environmental issue, PFOA-induced metabolic alterations have been reported in the lymphocytes in human peripheral blood [38] and in children with physician-diagnosed nonalcoholic fatty liver disease (NAFLD) [39]. However, an integrative analysis of the disturbance of the metabolome and gut microbiota by PFOA exposure has not been carried out. In this study, we not only revealed the changes in metabolites in serum, but also the metabolic alterations in the feces and liver. In addition, we performed an integrative analysis of fecal metabolites and the gut microbiota and revealed the association between them under the influence of PFOA exposure. The profiles of both fecal metabolites and the gut microbiota were disturbed by PFOA exposure.

The relative abundance of *Roseburia*, *Tyzzerella*, *Eubacterium coprostanoligenes*, and *Intestinimonas* was reduced by PFOA exposure. *Roseburia* is considered a commensal bacterium with anti-inflammatory properties, which could restore beneficial gut microorganisms [40]. *Roseburia intestinalis* can maintain energy homeostasis and prevent gut inflammation [41]. Lower levels of *Tyzzerella* were found in patients with acute myocardial infarction and in patients with esophageal cancer [42,43]. *Tyzzerella* has been reported to be associated with the dietary intake of fatty acids [44]. The growth of *Tyzzerella* was inhibited by the combinatory supplementation of theabrownin and a high-sugar diet [45]. *E. coprostanoligenes* was correlated with the level of diacylglycerols [46]. A reduction in *E. coprostanoligenes* was inversely associated with serum aspartate transferase (AST) and ALT levels in high-fat-diet-fed mice [47]. *Intestinimonas* spp. has been detected in lysine-enriched stool dilutions, and *Intestinimonas*-like bacteria are known as butyrate producers, which utilize lysine and Nε-fructosyllysine in formula-fed infants and adults [48]. A reduction in these microorganisms might have an influence on the immune response and metabolic activity of the host.

The relative abundance of *Alistipes*, *Clostridium ASF 356*, *Clostridiales*, and *Lachnospiraceae UCG 004* was significantly enriched by PFOA exposure. *Alistipes* is a conditional pathogenic bacterium, and it was increased by high-fat diets and high-sucrose diets [49]. *Alistipes* was considered as a gut microbiota marker that was shared among obese patients with various metabolic disorders [50]. A higher abundance of *Alistipes* was found in patients with different brain-related diseases, including Alzheimer’s disease, attention deficit hyperactivity disorder, schizophrenia, autism spectrum disorder, major depressive disorder, Parkinson’s disease, and bipolar disorder [51]. In addition, *Alistipes* was isolated from patients with abdominal and rectal abscesses and was associated with depression and colorectal cancer [52]. As a bile-tolerant microorganism, the abundance of *Alistipes* could be increased by an animal-based diet [53]. The enrichment of *Alistipes* was also induced by the dietary supplementation of castalagin, which is an active compound in the polyphenol-rich berry camu-camu [54]. An in vitro study suggested that *Clostridium ASF 356* consumed isoleucine, valine, alanine, threonine, lactate, and other metabolites, and it was also involved in cross-feeding. Some species in *Clostridium* are pathogenic, such as *C. difficile*.

Bile acid metabolism was disrupted by PFOA exposure in this study. Bile acids are synthesized in the liver from cholesterol and stored in the gallbladder, and they are released into the intestine in response to a meal and then pass through the enterohepatic circulation. Before secretion, the primary bile acids are typically conjugated to taurine and glycine. Primary bile acids, cholic acid and chenodeoxycholic acid, can be converted into secondary bile acids through bile acid deconjugation and 7-alpha hydroxylation. The gut microbiota can regulate bile acid metabolism through the reduction of tauro-β-muricholic acid levels, which is a nuclear receptor farnesoid X receptor (FXR) antagonist [55]. The synthesis of bile acids is under negative feedback control in the liver and ileum through FXR activation [55]. An association between serum bile acids and both serum PFOA and fetal growth endpoints was found in a cohort of 313 pregnant African American women [56]. Increased circulating bile acids due to a mixture of five PFAS, which consisted of PFOA, PFOS, PFNA, PFHxS, and GenX, were observed in C57BL/6J mice [57]. Age-related leaky gut and inflammation could be ameliorated by probiotic cocktail supplementation by increasing the activity of bile salt hydrolase, leading to an increase in taurine that stimulated tight junctions [58]. The deficiency of secondary bile acids induced by dysbiosis could promote intestinal inflammation, which could be alleviated through the supplementation of secondary bile acids in mouse models [59].

Impairment of the gene expression involved in bile acid metabolism by PFOA was reported in human HepaRG hepatoma cells. In particular, CYP7A1, the key enzyme catalyzing the rate-limiting step of bile acid synthesis, was decreased [60]. In addition to enzymes involved in bile acid biosynthesis, the expression levels of genes related to bile acid transport, *Bsep* and *Mrp2*, were also reduced by PFOA in a 3D primary mouse liver spheroid model [61]. Moreover, PFOA has been reported to interact with the human bile acid transporter Na^+^/taurocholate co-transporting polypeptide [62]. PFOA reduced the mRNA and protein expression of organic anion transporting polypeptides1a1, 1a4, and 1b2 in a mouse model through the activation of peroxisome proliferator-activated receptor (PPAR) alpha; these are the major transporters responsible for the uptake of bile acids into the liver [63].

Alterations of tryptophan metabolism were the most reported PFAS-associated metabolic signature in human studies [64]. The gut microbes can transform tryptophan into indole and its derivatives, such as 3-indoleacrylic acid and 3-indoleacetic acid, the level of which was reduced by PFOA exposure in this study. In particular, 3-indoleacrylic acid has been reported to promote intestinal epithelial barrier function and suppress inflammatory responses [65]. The genetic capacity of gut microorganisms utilizing mucins and metabolizing tryptophan is reduced in patients with inflammatory bowel disease [65]. As a cytoplastic receptor, AhR is an important factor in tissue homeostasis and immunity, which allows the adaption of immune cells to environmental conditions [66]. Moreover, 3-indoleacetic acid was reported as one of the dominant activators of AhR in the cecal content of mouse and stool samples from human participants [67]. In addition, 3-indoleacetic acid could promote intestinal barrier integrity and suppress inflammatory responses through the activation of the AhR transcription factor, which further promoted AhR-dependent IL-22 transcription [68]; 3-indoleacetic acid is also the activator of the pregnane X receptor (PXR), which induces IL-35^+^ B cell generation together with lipopolysaccharide through PXR and Toll-like receptor 4 [69]. Furthermore, 3-indoleacetic acid has beneficial effects such as alleviating ankylosing spondylitis [70]; 3-indoleacetic acid was significantly reduced in mice fed a high-fat diet [71]. Supplementation of 3-indoleacetic acid could alleviate nonalcoholic fatty liver disease through the attenuation of the inflammatory response, oxidative stress, and hepatic lipogenesis [72]. Moreover, 3-indoleacetic acid influenced chemotherapy efficacy in patients with pancreatic cancer in two independent pancreatic ductal adenocarcinoma cohorts [73]. In a pancreatic ductal adenocarcinoma mouse model, fecal microbiota transplantation and the supplementation of 3-indoleacetic acid could also enhance the chemotherapeutic efficacy in humanized gnotobiotic mice [73]. Along with the reduction of 3-indoleacrylic acid and 3-indoleacetic acid by PFOA, these beneficial effects might be compromised.

This study had several limitations. Firstly, this study only included male mice, while female mice were not included. A gender-balanced study design that includes both male mice and female mice could improve this area of study. Second, the PFOA level in the drinking water that control mice received was not detected. Considering the wide distribution of PFOA, there was a probability that the drinking water that the control mice received contained low levels of PFOA. The PFOA level in drinking water was reported to be between 20 and 70 ng/L in Georgia, US [74]. In order to test the toxic effect of PFOA on gut-microbiota-related metabolites, the exposure level in this study was set to 1 ppm, which is higher than the environmentally relevant level. The dose needs to be lowered in future studies to explore the health effects of PFOA at environmentally relevant levels.

## 5. Conclusions

PFOA has become a serious public health concern recently. In the present study, PFOA disturbed both the gut microbiota composition and the metabolic profiles of the feces, serum, and liver in mice. A correlation was found between the gut microbiota and different fecal metabolites. PFOA exposure induced significant alterations of gut-microbiota-related metabolites, including bile acids and tryptophan metabolites, such as 3-indoleacrylic acid and 3-indoleacetic acid. Our findings could improve the understanding of the health effects of PFOA mediated through the gut microbiota.

## Data Availability

The data are available upon reasonable request. The data are not publicly available due to privacy.

## Supplementary Materials

The following supporting information can be downloaded at: https://www.mdpi.com/article/10.3390/metabo13060707/s1, Figure S1: PLS-DA plot for fecal metabolites.
