# Supplementary figures and images for: Effects of Perfluorooctanoic Acid on Gut Microbiota and Microbial Metabolites in C57BL/6J Mice
Source: Metabolites. 2023 May 30;13(6):707. doi: 10.3390/metabo13060707 (PMC10305235; doi:10.3390/metabo13060707)

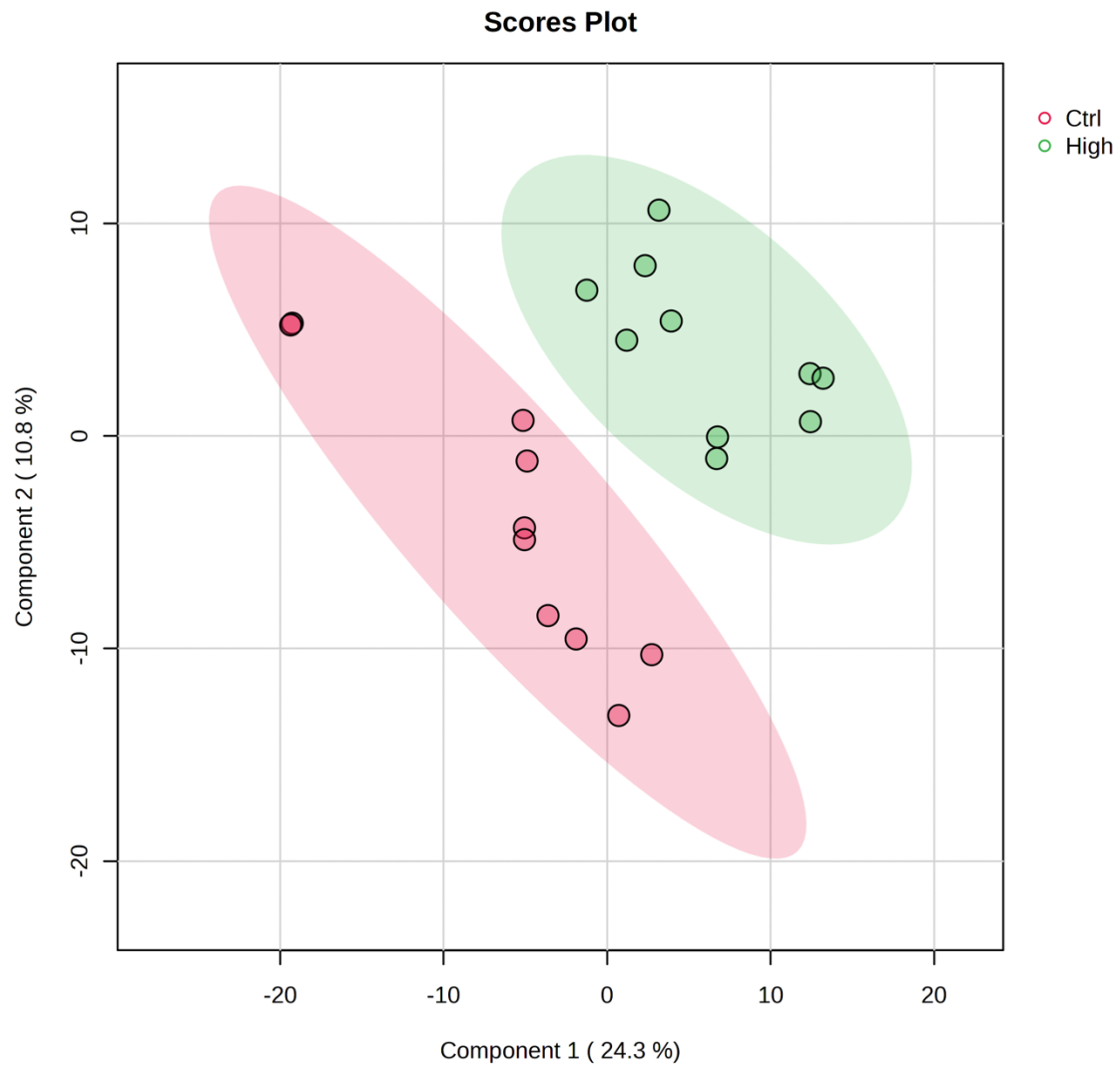

Figure S1. PLS-DA plot for fecal metabolites

Supplement: Supplementary file 1 [file metabolites-13-00707-s001.zip › metabolites-2391158-supplementary.pdf]
